# Impact of Chronic BDNF Depletion on GABAergic Synaptic Transmission in the Lateral Amygdala

**DOI:** 10.3390/ijms20174310

**Published:** 2019-09-03

**Authors:** Susanne Meis, Thomas Endres, Thomas Munsch, Volkmar Lessmann

**Affiliations:** 1 Institut für Physiologie, Otto-von-Guericke-Universität, D-39120 Magdeburg, Germany (T.E.) (T.M.); 2 Center for Behavioral Brain Sciences, D-39106 Magdeburg, Germany

**Keywords:** amygdala, GABA, BDNF, heterozygous BDNF knockout mice, LTP

## Abstract

Brain-derived neurotrophic factor (BDNF) has previously been shown to play an important role in glutamatergic synaptic plasticity in the amygdala, correlating with cued fear learning. While glutamatergic neurotransmission is facilitated by BDNF signaling in the amygdala, its mechanism of action at inhibitory synapses in this nucleus is far less understood. We therefore analyzed the impact of chronic BDNF depletion on GABA_A_-mediated synaptic transmission in BDNF heterozygous knockout mice (BDNF^+/−^). Analysis of miniature and evoked inhibitory postsynaptic currents (IPSCs) in the lateral amygdala (LA) revealed neither pre- nor postsynaptic differences in BDNF^+/−^ mice compared to wild-type littermates. In addition, long-term potentiation (LTP) of IPSCs was similar in both genotypes. In contrast, facilitation of spontaneous IPSCs (sIPSCs) by norepinephrine (NE) was significantly reduced in BDNF^+/−^ mice. These results argue against a generally impaired efficacy and plasticity at GABAergic synapses due to a chronic BDNF deficit. Importantly, the increase in GABAergic tone mediated by NE is reduced in BDNF^+/−^ mice. As release of NE is elevated during aversive behavioral states in the amygdala, effects of a chronic BDNF deficit on GABAergic inhibition may become evident in response to states of high arousal, leading to amygdala hyper-excitability and impaired amygdala function.

## 1. Introduction

Brain-derived neurotrophic factor (BDNF) signaling via its cognate TrkB (tropomyosin-related kinase B) receptor regulates differentiation and survival during neuronal maturation. Furthermore, BDNF plays a pivotal role in synaptic strength and plasticity, and is thereby essential for learning and memory [1,2,3,4,5]. While glutamatergic transmission is, in many brain areas, facilitated by acute or chronic actions of BDNF via TrkB receptors, the role of BDNF signaling at inhibitory synapses seems to be more diverse [1,6,7]. Acute application of BDNF often leads to reduced GABAergic neurotransmission [8,9,10,11], but opposite effects [12,13,14] and biphasic temporal modulation [6,15] have also been described. Chronic BDNF depletion resulted in increased inhibitory synaptic activity in the dentate gyrus and superior colliculus [16,17], while GABAergic inhibitory function was strongly impaired in the visual cortex and in thalamic circuits [18,19].

In the amygdala, inhibitory mechanisms are now recognized to contribute significantly to fear learning and extinction [20]. GABAergic synaptic transmission seems to be involved in rhythmic activity, supporting the interaction of the amygdala with other brain structures during the retrieval of fear memory [21]. Reduction of GABAergic tone may facilitate fear generalization, as well as the development of anxiety disorders [22,23]. In line with this notion, amygdala hyper-excitability is a common phenomenon in disorders like epilepsy, anxiety, and stress-related diseases [24]. In addition, inhibitory control of the amygdala is regulated by different neuromodulatory systems. In the basal amygdala (BA), GABA release is facilitated via presynaptic α1A adrenergic receptors. This mechanism, regulating neuronal excitability in the BA, is severely impaired by stress and attenuated after fear conditioning [25,26].

A critical role for BDNF signaling in amygdala-dependent fear learning, as well as glutamatergic synaptic plasticity, was substantiated by several recent studies (for review and references, see [27,28]). Indeed, long-term potentiation (LTP) at glutamatergic thalamic afferents to the lateral amygdala (LA) was prevented by acute inhibition of BDNF/TrkB signaling [29]. Moreover, learning-induced long-term changes at cortico-LA synapses were absent in heterozygous BDNF^+/−^ mice in parallel with a deficit in fear memory consolidation [30]. As reported previously, BDNF ELISA analysis in the basolateral amygdala in 4- to 5-week-old BDNF^+/−^ mice showed a reduction of BDNF protein levels to around 50% compared to wild-type (WT) littermates [29]. However, information about the influence of BDNF signaling on GABAergic mechanisms in the amygdala is scarce. In amygdala neuronal cell cultures, acute BDNF treatment resulted in rapid internalization of surface GABA_A_Rα1. This TrkB-dependent internalization of GABA_A_Rs was hypothesized to partially underlie a transient period of enhanced amygdala activation during fear memory consolidation [31,32]. However, the effect of chronically decreased BDNF levels on GABAergic inhibitory circuits in the amygdala remains elusive. Therefore, in this study, we used patch clamp electrophysiological recordings in an in vitro slice preparation of heterozygous BDNF^+/−^ mice and their wild-type littermates to analyze GABAergic synaptic inputs to LA projection neurons. Our results indicate that chronic BDNF reduction in the amygdala of BDNF^+/−^ mice to about 50% of WT levels [29,33] did neither impair basal synaptic GABAergic transmission nor synaptic plasticity of inhibitory GABAergic inputs (iLTP). However, positive modulation of GABAergic synaptic transmission by norepinephrine (NE) was significantly reduced in BDNF^+/−^ mice, which may lead to reduced GABAergic tone and hyper-excitability in the amygdala during emotionally significant events.

## 2. Results

### 2.1. Basal GABAergic Synaptic Transmission

**Figure 1:**
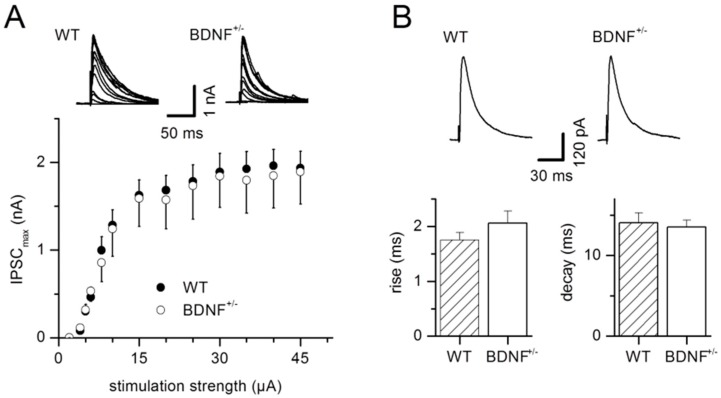
Basal synaptic efficacy and inhibitory postsynaptic current (IPSC) kinetics are unchanged in brain-derived neurotrophic factor heterozygous knockout (BDNF^+/−^) mice. (**A**) Input–output relationship, measuring IPSC amplitudes as a function of stimulus intensity, displayed no change between the two experimental groups. Traces depict IPSC amplitudes with rising stimulation strength for a representative wild-type (WT) or BDNF^+/−^ neuron, respectively. WT: *n* = 9 neurons from 9 animals, BDNF^+/−^: *n* = 8 neurons from 6 animals. (**B**) Neither rise time nor decay time constants (τ) of evoked IPSCs differed between genotypes. Typical evoked IPSCs of a WT and BDNF^+/−^ lateral amygdala (LA) projection neuron, respectively, are shown at the top. WT: *n* = 9 neurons from 5 animals, BDNF^+/−^: *n* = 9 neurons from 6 animals.

We analyzed input–output relationships to test for potential BDNF-dependent alterations in inhibitory synaptic efficacy in LA projection neurons. GABAergic inhibitory postsynaptic currents (IPSCs) arising from local inhibitory interneurons were isolated in the presence of DNQX and AP5 to block glutamatergic transmission. Evoked IPSC (eIPSC) amplitudes as a function of afferent stimulus intensity were unchanged when comparing BDNF^+/−^ mice with their wild-type littermates (WT: *n* = 9, BDNF^+/−^: *n* = 8; factor genotype: F_1,195_ = 0.26, *p* = 0.61; interaction of factors genotype × stimulation strength: F_12,195_ = 0.04, *p* = 1, Figure 1A). Increasing stimulation strength led to increased eIPSC amplitudes, as expected (factor stimulation strength: F_12,195_ = 21.85, *p* < 0.0001, Figure 1A). In addition, we examined the kinetics of eIPSCs. Neither the rise time (10–90%; WT: 1.76 ± 0.13 ms, *n* = 9; BDNF^+/−^: 2.06 ± 0.22 ms, *n* = 9; *p* = 0.26) nor the decay time constant (τ; WT: 14.1 ± 1.2 ms, *n* = 9; BDNF^+/−^: 13.5 ± 0.9 ms, *n* = 9; *p* = 0.93) of eIPSCs was different between genotypes (Figure 1B).

**Figure 2:**
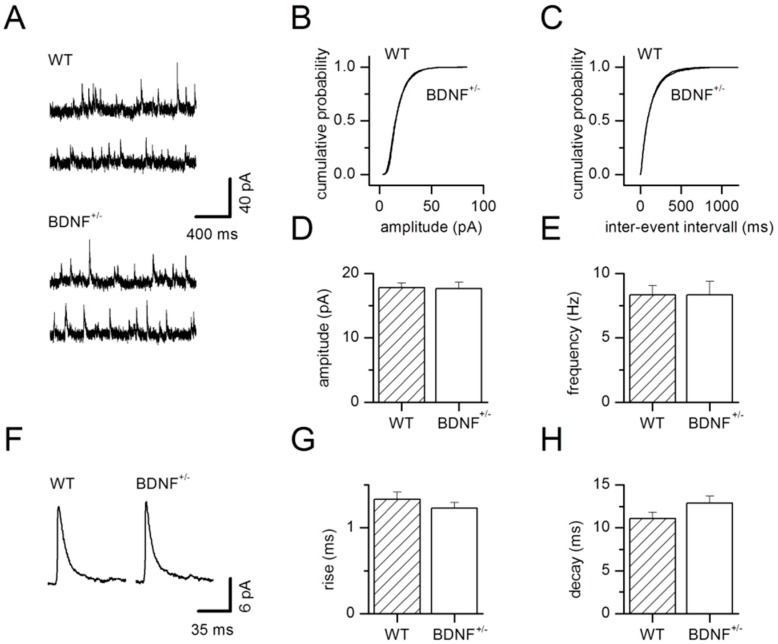
Properties of miniature GABAergic IPSCs (mIPSCs) in BDNF^+/−^ and WT mice. (**A**) Representative examples of mIPSCs recorded in LA projection neurons of WT and BDNF^+/−^ mice. (**B**) Cumulative amplitude and (**C**) inter-event interval histograms obtained from WT and BDNF^+/−^ neurons, with 300 events analyzed for each individual cell. Mean mIPSC amplitude (**D**) and frequency (**E**) pooled for WT and BDNF^+/−^ neurons. Note the unaltered amplitude (**B**,**D**) and frequency (**C**,**E**) of mIPSCs in BDNF^+/−^ mice compared to WT littermates. (**F**) Typical examples of averaged mIPSCs for a representative WT or BDNF^+/−^ neuron, respectively. Neither rise time (**G**) nor decay time constant τ (**H**) of mIPSCs differed between genotypes. WT: *n* = 10 neurons from 10 animals, BDNF^+/−^: *n* = 8 neurons from 8 animals.

To check for possible pre- and postsynaptic changes at inhibitory synapses, we recorded miniature IPSCs (mIPSCs). TTX (1 µM) was added to the bath solution to abolish action potential-evoked transmitter release. Representative examples of recorded mIPSCs for wild-type and BDNF^+/−^ neurons are shown in Figure 2A. For cumulative amplitude (Figure 2B) and inter-event interval histograms (Figure 2C), 300 events per neuron were analyzed for all wild-type or BDNF^+/−^ neurons, respectively. Mean mIPSC amplitudes were unchanged in BDNF^+/−^ mice compared to WT littermate controls (WT: 17.8 ± 0.7 pA, *n* = 10; BDNF^+/−^: 17.6 ± 1.0 pA, *n* = 8; *p* = 0.97, Figure 2D). Likewise, analysis of mIPSC frequency revealed no differences in basal presynaptic GABA release in BDNF^+/−^ mice (WT: 8.3 ± 0.7 Hz, *n* = 10; BDNF^+/−^: 8.4 ± 1.0 Hz, *n* = 8; *p* = 0.90, Figure 2E). Averaged mIPSCs for wild-type and BDNF^+/−^ neurons are shown in Figure 2F. Rise as well as decay kinetics of mIPSCs (Figure 2G,H) were alike in the two genotypes (rise 10–90%; WT: 1.33 ± 0.08 ms, *n* = 10; BDNF^+/−^: 1.23 ± 0.07 ms, *n* = 8; *p* = 0.51; decay τ; WT: 11.1 ± 0.7 ms, *n* = 10; BDNF^+/−^: 12.9 ± 0.8 ms, *n* = 8; *p* = 0.12).

**Figure 3:**
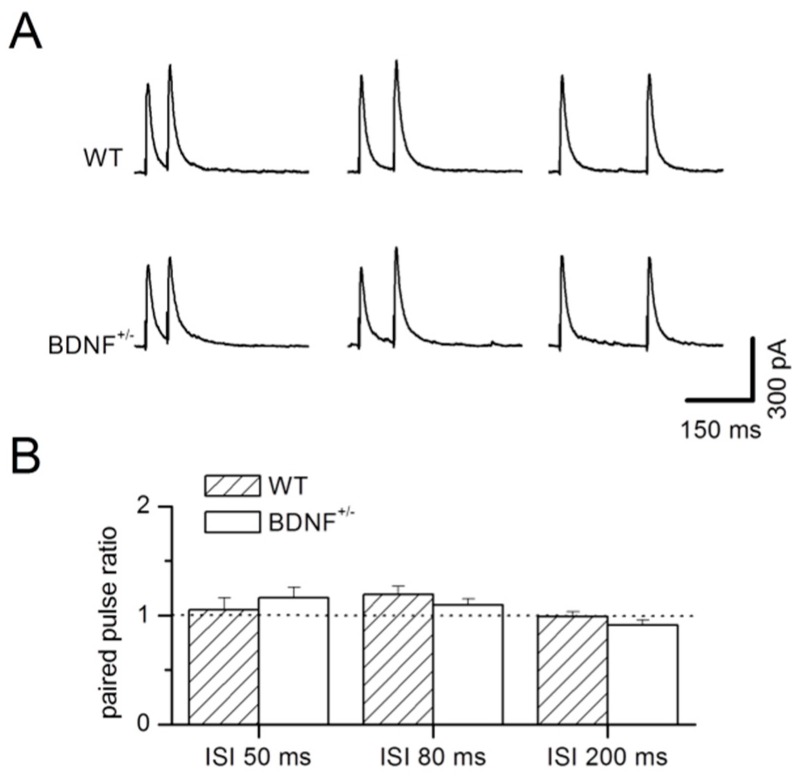
Paired-pulse ratio (PPR) of GABA_A_ mediated evoked IPSCs in LA projection neurons are unaltered in BDNF^+/−^ mice. (**A**) Typical current traces of evoked paired synaptic stimulations at different inter-stimulus intervals (ISIs) in a representative WT neuron (top) and BDNF^+/−^ neuron (bottom), respectively. (**B**) Mean PPRs at different ISIs (as indicated). Note the unaltered PPR in BDNF^+/−^ mice compared to WT littermates. WT: *n* = 10 neurons from 6 animals, BDNF^+/−^: *n* = 10 neurons from 6 animals.

To further address possible alterations in the efficacy of presynaptic transmitter release by the chronic BDNF deficit, we examined paired-pulse facilitation of eIPSCs. Paired-pulse ratios (PPRs) were calculated as amplitude of the second IPSC divided by the first at three different inter-stimulus intervals (ISIs), but did not reveal any difference between BDNF^+/−^ and WT littermates (factor genotype: F_1,54_ = 0.12, *p* = 0.73; interaction of the factors genotype × PPR: F_2,54_ = 1.12, *p* = 0.33, Figure 3A,B), while an effect for the factor PPR was determined (factor PPR: F_2,54_ = 3.65, *p* = 0.03). For better comparison, stimulation parameters were adjusted such that the mean amplitudes of the first IPSC were alike in both genotypes (ISI 50 ms; WT: 463.3 ± 53.7 pA, *n* = 10; BDNF^+/−^: 456.5 ± 24.4 pA, *n* = 10; *p* = 0.97; ISI 80 ms; WT: 450.5 ± 64.3 pA, *n* = 10; BDNF^+/−^: 444.7 ± 23.9 pA, *n* = 10; *p* = 0.63; ISI 200 ms; WT: 429.8 ± 66.9 pA, *n* = 10; BDNF^+/−^: 475.6 ± 21.2 pA, *n* = 10; *p* = 0.28).

**Figure 4:**
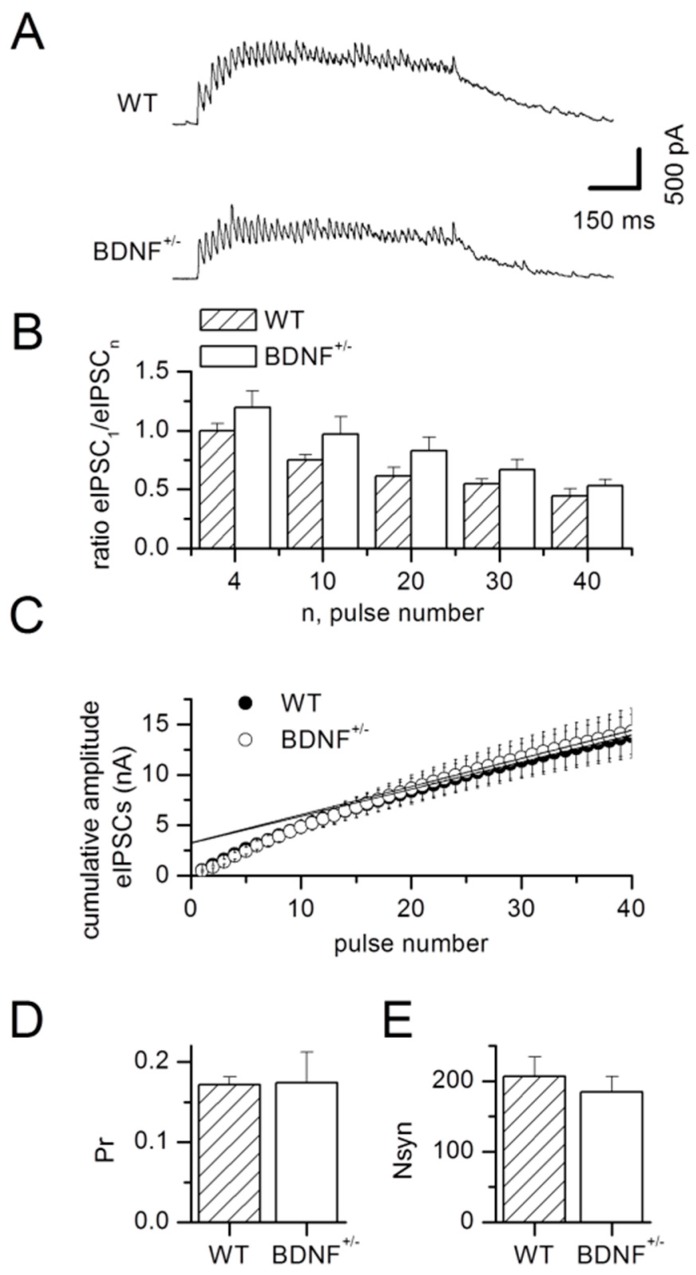
Unaltered synaptic fatigue of evoked IPSCs in response to high frequency synaptic stimulation in BDNF^+/−^ mice. (**A**) Examples of evoked IPSCs during repetitive synaptic stimulation at 40 Hz for 1 s in LA neurons of WT (top) and BDNF^+/−^ (bottom) mice. (**B**) Synaptic fatigue was quantified as mean ratio of IPSC amplitudes evoked by the 4th, 10th, 20th, 30th, and 40th stimulus normalized to the IPSC amplitude evoked by the first stimulus. No change in synaptic fatigue was detected in WT versus BDNF^+/−^ mice. WT: *n* = 10 neurons from 6 animals, BDNF^+/−^: *n* = 10 neurons from 5 animals. (**C**) Cumulative amplitude of evoked IPSCs (eIPSCs) was plotted against number of stimuli to obtain the readily releasable pool (RRP) by extrapolation back to the y-axis. Neither RRP nor (**D**) release probability (Pr) nor (**E**) the number of synaptic release sites activated by the stimulus (Nsyn) were altered between genotypes. WT: *n* = 10 neurons from 6 animals, BDNF^+/−^: *n* = 10 neurons from 5 animals.

Presynaptic efficacy was further examined by trains of stimulation at a frequency of 40 Hz for 1 s. As expected, we observed synaptic fatigue under these conditions, as calculated for the mean ratios of current amplitudes of the 4th, 10th, 20th, 30th, and 40th IPSC in relation to the first IPSC peak amplitude (factor pulse number in the train: F_4,90_ = 13.76, *p* < 0.0001, Figure 4A,B). Even though the ANOVA revealed a general effect of the factor genotype (F_1,90_ = 8.70, *p* = 0.004), the synaptic fatigue was not influenced by the genotype of the animals (interaction of factors genotype × pulse number in the train: F_4,90_ = 0.21, *p* = 0.93, Figure 4A,B).

Stimulation parameters were adjusted to elicit similar peak amplitudes for the first IPSC in the train in both genotypes (WT: 537.1 ± 72.4 pA, *n* = 10; BDNF^+/−^: 478.1 ± 71.5 pA, *n* = 10; *p* = 0.58). From these experiments, we also quantified (compare [18]) the readily releasable pool (RRP, Figure 4C), the release probability (Pr, Figure 4D), and the number of synaptic release sites activated by the stimulus (Nsyn, Figure 4E). None of these parameters were altered in BDNF^+/−^ mice compared to WT littermates (RRP: WT: 3280.1 ± 528.6 pA, *n* = 10; BDNF^+/−^: 3274.4 ± 525.3 pA, *n* = 10; *p* = 0.91; Pr: WT: 0.17 ± 0.01, *n* = 10; BDNF^+/−^: 0.17 ± 0.04, *n* = 10; *p* = 0.12; Nsyn: WT: 206.5 ± 27.9, *n* = 10; BDNF^+/−^: 184.3 ± 22.5, *n* = 10; *p* = 0.58).

These results argue against any presynaptic or postsynaptic alteration in the efficacy of GABAergic synaptic transmission in BDNF^+/−^ mice compared to WT littermates.

### 2.2. LTP at GABAergic Synapses (iLTP)

**Figure 5:**
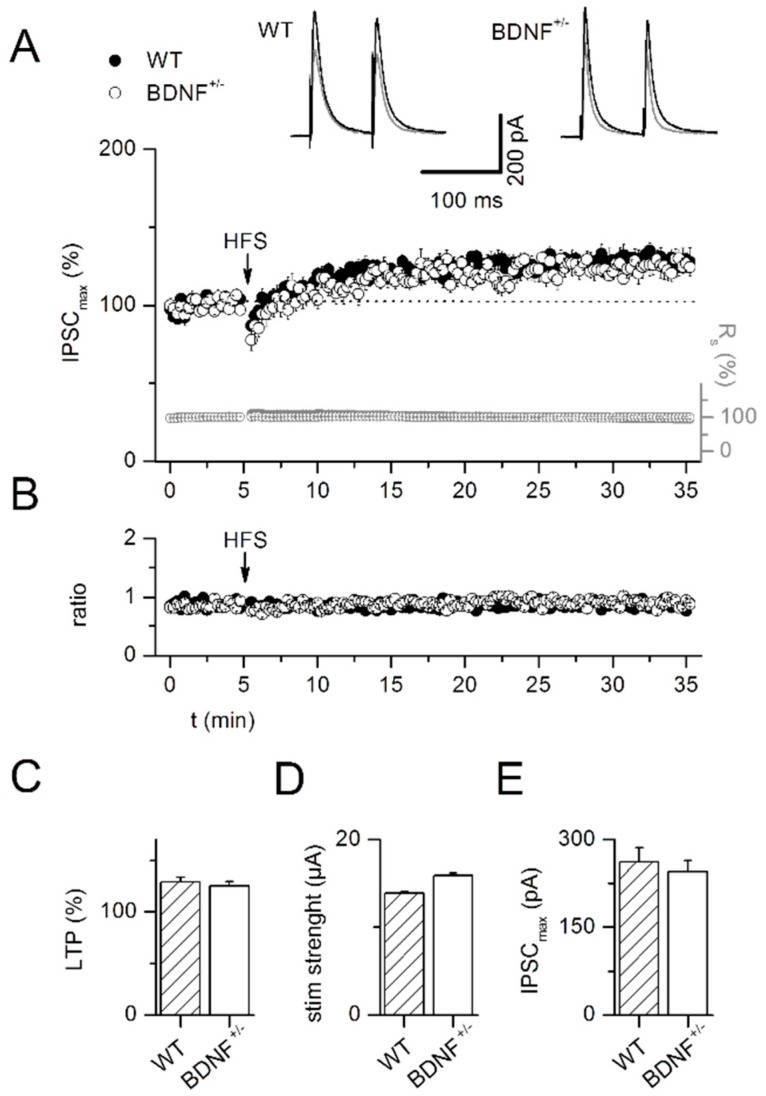
Intact inhibitory long-term potentiation (iLTP) of GABAergic synaptic inputs to LA projection neurons in BDNF^+/−^ mice. ILTP was induced by pairing afferent stimulation in the LA (100 Hz, 1 s) with postsynaptic depolarization to −10 mV. (**A**) Time course of averaged evoked IPSCs and series resistance in all neurons recorded from WT and BDNF^+/−^ mice, respectively. In both genotypes, long-term potentiation (LTP) could be reliably induced. Insets depict averaged eIPSCs 5 min before LTP induction and during the last 5 min of recordings for WT and BDNF^+/−^ mice, respectively. (**B**) Paired-pulse ratio was unchanged during the time course of the experiment. (**C**) Summary of LTP expressed in the two genotypes. (**D**,**E**) Stimulus intensity was adjusted to elicit synaptic responses with amplitudes of 150–200 pA to control for identical induction conditions in both genotypes. Neither stimulation strength (**D**) nor amplitude of the baseline IPSCs (**E**) differed between genotypes. WT: *n* = 10 neurons from 6 animals, BDNF^+/−^: *n* = 9 neurons from 7 animals.

Experiments were conducted in the presence of DNQX to block non-NMDA glutamatergic synaptic transmission. Pairing of afferent stimulation (100 Hz, 1 s) with postsynaptic depolarization to −10 mV induced inhibitory long-term potentiation (iLTP) of IPSCs in LA projection neurons. We observed iLTP as an increase in average IPSC amplitude at 30 min after the induction protocol compared to baseline levels in slices from wild-type mice as well as BDNF^+/−^ mice (WT: 128.6 ± 4.8%, *n* = 10; BDNF^+/−^: 125.1 ± 4.1%, *n* = 9; factor phase: F_1,34_ = 70.15, *p* < 0.0001, Figure 5A,C). ILTP was comparable in both experimental groups (factor genotype: F_1,34_ = 0.33, *p* = 0.57; interaction of the factors genotype × phase: F_1,34_ = 0.29, *p* = 0.59). Neither stimulation strength (WT: 13.9 ± 1.3 µA, *n* = 10; BDNF^+/−^: 15.9 ± 3.0 µA, *n* = 9; *p* = 1, Figure 5D) nor amplitude of the baseline IPSC (WT: 261.4 ± 24.5 pA, *n* = 10; BDNF^+/−^: 245.3 ± 19.1 pA, *n* = 9; *p* = 0.84, Figure 5E) differed between genotypes. ILTP induction did not influence paired-pulse ratio (WT; baseline: 0.89 ± 0.04, LTP: 0.86 ± 0.03, *n* = 10, *p* = 0.23; BDNF^+/−^; baseline: 0.85 ± 0.04, LTP: 0.89 ± 0.02, *n* = 9, *p* = 0.57, Figure 5B) in either genotype (*p* = 0.45).

In addition, iLTP at GABAergic synapses was independent of NMDA receptor activation, as similar potentiation could be observed when DNQX and the NMDA receptor antagonist AP5 were co-applied (122.4 ± 5.3%, *n* = 9, *p* = 0.32).

These results indicate that plasticity of GABAergic IPSCs under our recording conditions may be expressed at the postsynaptic site as suggested by Bauer and LeDoux [34], and that BDNF^+/−^ mice do not show altered plasticity at inhibitory synapses in LA projection neurons.

### 2.3. Glutamatergic Drive onto LA Interneurons

Besides direct changes at GABAergic synapses, chronic BDNF reduction could modify inhibitory synaptic transmission indirectly through actions on the excitatory glutamatergic input to local interneurons. To test for such changes of excitatory drive on interneurons, we recorded spontaneous IPSCs (sIPSCs) in LA projection neurons before and after combined application of the glutamate receptor antagonists AP5 and DNQX. Application of these inhibitors did neither induce a significant shift in mean amplitudes nor in mean frequencies of sIPSCs in both genotypes, demonstrating a lack of spontaneous glutamatergic drive on local interneurons under our recording conditions (amplitude: WT control 20.3 ± 0.7 pA, AP5/DNQX 19.8 ± 0.6 pA, *n* = 8, *p* = 0.20; BDNF^+/−^ control 19.6 ± 1.1 pA, AP5/DNQX 18.9 ± 1.4 pA, *n* = 8, *p* = 0.20; frequency: WT control 12.9 ± 1.9 Hz, AP5/DNQX 12.7 ± 2.0 Hz, *n* = 8, *p* = 0.67; BDNF^+/−^ control 10.1 ± 1.2 Hz, AP5/DNQX 9.3 ± 1.1 Hz, *n* = 8, *p* = 0.30). Furthermore, neither sIPSC amplitudes (*p* = 0.80) nor frequencies (*p* = 0.31) differed between the two experimental groups. Therefore, interneuron excitability seems to be unchanged by chronic BDNF depletion to ~50% of WT values in BDNF^+/−^ mice [29,33].

### 2.4. Modulation of sIPSCs by Norepinephrine (NE)

**Figure 6:**
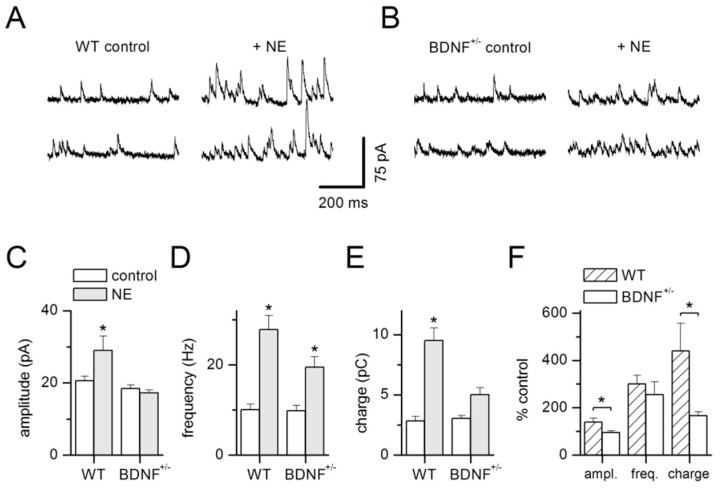
Norepinephrine (NE)-dependent stimulation of GABA-mediated synaptic activity in WT and BDNF^+/−^ mice. Spontaneous IPSCs (sIPSCs) recorded from the same representative WT (**A**) and BDNF^+/−^ (**B**) projection neuron, respectively, before and after application of NE (10 µM). (**C**) sIPSC amplitude, (**D**) frequency, and (**E**) charge pooled during control conditions and after addition of NE demonstrates a significant increase in sIPSC amplitude in WT mice only, while frequency of sIPSCs is augmented significantly in both genotypes. (**F**) Increase in sIPSC amplitude and charge was significantly different in WT versus BDNF^+/−^ mice, while sIPSC frequency increase was similar in both genotypes. WT: *n* = 12 neurons from 7 animals, BDNF^+/−^: *n* = 13 neurons from 6 animals. * *p* < 0.05, Tukey post-hoc comparisons.

Norepinephrine (NE) is known to facilitate sIPSCs in the basal amygdala of rats and mice [35,36,37,38] In our experiments in the lateral amygdala, application of 10 μM NE also strongly increased the frequency of sIPSCs in BDNF^+/−^ and WT littermate neurons, while sIPSC amplitudes were only affected in WT mice (Figure 6). Current traces under control conditions and during maximal NE effect (Figure 6A,B) illustrate sIPSCs in the same representative WT (Figure 6A) or BDNF^+/−^ (Figure 6B) projection neuron, respectively. Mean results for both genotypes are depicted in Figure 6C–F.

NE application resulted in increased sIPSC frequency in BDNF^+/−^ and WT littermate neurons (WT control: 10.1 ± 1.2 Hz, NE: 27.8 ± 3.1 Hz, *n* = 12; BDNF^+/−^ control: 9.8 ± 1.2 Hz, NE: 19.5 ± 2.3 Hz, *n* = 13; Figure 6D,F; factor NE treatment: F_1,46_ = 42.47, *p* < 0.0001) with an effect of genotype (factor genotype: F_1,46_ = 4.17, *p* = 0.04). ANOVA revealed a clear tendency for a different strength in sIPSC frequency modulation by NE between genotypes (interaction genotype × NE treatment: F_1,46_ = 3.59, *p* = 0.06). In contrast, sIPSC amplitudes were only increased in WT mice (WT control: 20.6 ± 1.3 pA, NE: 29.0 ± 4.0 pA, *n* = 12; BDNF^+/−^ control: 18.5 ± 1.0 pA, NE: 17.3 ± 0.8 pA, *n* = 13, Figure 6C,F; factor genotype: F_1,46_ = 10.53, *p* = 0.002; factor NE treatment: F_1,46_ = 2.83, *p* = 0.1; interaction of factors genotype × NE treatment: F_1,46_ = 5.13, *p* = 0.03).

We quantified charge transfer carried by the sIPSCs in both genotypes (WT control: 2.9 ± 0.4 pC, NE 9.5 ± 1.1 pC, *n* = 12; BDNF^+/−^ control: 3.1 ± 0.2 pC, NE: 5.0 ± 0.6 pC, *n* = 13, Figure 6E,F). Indeed, charge transfer increased significantly upon addition of NE (factor NE treatment: F_1,46_ = 46.4, *p* < 0.0001). This effect was significantly enhanced in WT compared to BDNF^+/−^ mice (factor genotype: F_1,46_ = 11.48, *p* = 0.002; interaction of factors genotype × NE treatment: F_1,46_ = 14.03, *p* = 0.001).

**Figure 7:**
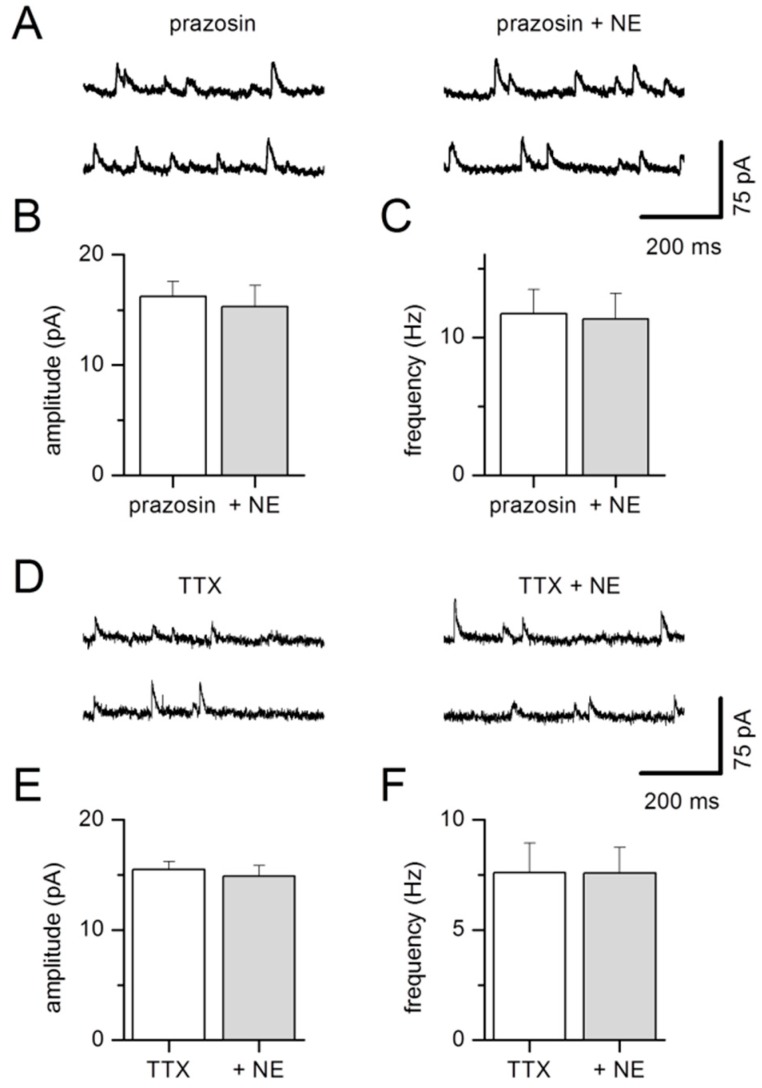
NE effects on GABA-mediated synaptic activity are blocked by the α1-adrenergic antagonist prazosin (**A**–**C**) or in the presence of TTX (**D**–**F**). (**A**) Examples of sIPSCs recorded in an individual LA projection neuron before (left) and during application of NE in the continuous presence of prazosin (right). (**B**) sIPSC amplitude and (**C**) frequency pooled during control conditions and after addition of NE are not increased in the presence of prazosin, *n* = 7 neurons from 6 animals. (**D**) Examples of mIPSCs recorded in an individual projection neuron before (left) and during application of NE in the continuous presence of TTX (right). No effect of NE was detected on mean mIPSC amplitudes (**E**) and mean mIPSC frequencies (**F**) between control conditions (TTX) and after addition of NE (TTX + NE), *n* = 8 neurons from 6 animals.

In the presence of the α1-adrenergic antagonist prazosin, neither amplitudes nor frequencies of sIPSCs recorded in LA projection neurons were altered by NE (mean sIPSC amplitude, prazosin 16.2 ± 1.3 pA; prazosin/NE 15.3 ± 1.9 pA *n* = 7, *p* = 0.44; mean sIPSC frequency, prazosin 11.7 ± 1.8 Hz; prazosin/NE 11.3 ± 1.9 Hz *n* = 7, *p* = 0.47, Figure 7A,B). Moreover, mIPSCs that were recorded in LA projection neurons in the presence of TTX were also not modified in the presence of NE (mean mIPSC amplitude, control 15.5 ± 0.7 pA; NE 14.9 ± 0.9 pA, *n* = 8, *p* = 0.12; mean mIPSC frequency, control 7.6 ± 1.3 Hz; NE 7.6 ± 1.2 Hz *n* = 8, *p* = 1, Figure 7C,D). These data indicate that in the LA, NE increases sIPSCs via activation of α1-adrenergic receptors, similar to results for the BA as previously reported [38]. Since addition of TTX abolished the NE effect, excitation of interneurons and subsequent spike propagation seems to be required for the observed NE modulation of sIPSCs. The reduced facilitation of sIPSCs by NE in BDNF^+/−^ mice might lead to increased excitability of LA projection neurons.

## 3. Discussion

In the present study, we focused on GABAergic synapses on LA projection neurons. Therefore, glutamatergic inputs were blocked with DNQX and AP5, and the stimulation electrode was placed within the LA to directly activate local interneurons. Chronic BDNF reduction in the LA of BDNF^+/−^ mice to about 50% of WT values [29] did neither impair basal synaptic GABAergic transmission nor inhibitory synaptic plasticity. However, positive modulation of interneuron activity by noradrenaline was significantly reduced by BDNF haplo-insufficiency, suggesting a role of BDNF signaling in regulating neuromodulatory transmitter effects in inhibitory synaptic circuits of the LA.

### 3.1. Basal GABAergic Transmission in BDNF^+/−^ Mice

Chronic BDNF reduction in BDNF^+/−^ mice has been shown previously to yield opposite effects on GABAergic synaptic transmission in different brain areas. In the dentate gyrus of the hippocampus, BDNF^+/−^ mice showed increased inhibitory synaptic activity accompanied by decreased excitability of granule cells. As the frequency of mIPSCs and paired-pulse depression of eIPSCs were both enhanced, the increased inhibition most likely resulted from enhanced presynaptic release probability of GABA [16]. Moreover, a rise of sIPSC frequencies recorded in dentate gyrus granule cells of BDNF^+/−^ mice was suggested to be due to an increase in interneuron firing rates [39]. Furthermore, enhancement of GABAergic inhibition, as seen in the superior colliculus of homozygous BDNF knockout mice (BDNF^−/−^ mice), was suggested to result from postsynaptic increase in GABA_A_ receptor expression [17].

In contrast, GABAergic inhibitory function was strongly impaired by chronically reduced levels of BDNF in the visual cortex [18]. BDNF^+/−^ mice showed decreased frequency and amplitude of mIPSCs, as well as diminished paired-pulse depression. In line with altered presynaptic GABA release in BDNF^+/−^ mice, release probability, steady-state release, and synchronous release of GABA were decreased [18]. Consistently, frequency and amplitude of mIPSCs were also reduced in the thalamic ventrobasal nucleus of BDNF^+/−^ mice, while the decay time constant was prolonged. Evoked IPSCs showed no alteration in paired-pulse depression or synaptic fatigue compared to WT littermates. This reduced GABAergic transmission under conditions of chronic BDNF deficit suggests reduced presynaptic function and/or reduced number of functional GABAergic synaptic boutons [19]. Likewise, disruption of activity-dependent BDNF transcription was reported to impair inhibitory synaptic transmission also in prefrontal cortex pyramidal neurons [40].

In the present study, we observed neither increased nor decreased efficacy of GABAergic synaptic inputs on LA projection neurons in BDNF^+/−^ mice. These results resemble glutamatergic synaptic transmission in the amygdala, where neither intrinsic membrane properties of LA projection neurons, nor presynaptic glutamate release, nor postsynaptic membrane properties were affected in BDNF^+/−^ mice [29]. Different brain areas may therefore show differential susceptibility to BDNF depletion for the development and/or preservation of proper GABAergic synaptic function.

### 3.2. LTP at GABAergic Synapses in the LA of BDNF^+/−^ Mice

In the present study, we focused on iLTP of GABAergic synapses on projection neurons. All glutamatergic inputs in the LA were inhibited by addition of DNQX and AP5 and local interneurons were directly activated by focal electrical stimulation. This experimental paradigm was chosen to directly investigate synaptic plasticity at GABAergic synapses in the absence of interfering effects from glutamatergic network activity [34,41,42,43,44]. We observed stable LTP induced by pairing postsynaptic depolarization with high-frequency presynaptic stimulation (HFS) at 100 Hz. Evoked IPSCs showed no change in PPR 30 min after LTP induction, resembling results in the rat LA [34].

In amygdala cultures kept 2–3 weeks in vitro, application of exogenous BDNF led to a reduction in surface GABA_A_Rα1 within 5 min. This effect was TrkB receptor and PKC dependent [32]. We observed previously that LTP at the glutamatergic thalamic input to LA projection neurons is supported by acute endogenous BDNF signaling, in which BDNF may arise from thalamic or intra-amygdala sources [29]. It seems reasonable to assume that the same stimulation paradigm applied directly in the LA as in the present study may elicit BDNF release from the same afferents/PN somata. Therefore, we hypothesized that BDNF^+/−^ mice might show a substantial reduction in acutely-released BDNF, less internalization of GABA_A_ receptors, and hence larger GABA LTP. Nevertheless, no differences were found between WT and BDNF^+/−^ mice. Therefore, a chronic reduction of endogenous BDNF to around 50% does not seem to compromise GABA LTP in LA projection neurons.

### 3.3. Norepinephrine

In our experiments, application of NE strongly increased the frequency and amplitude of sIPSCs in WT projection neurons via activation of α1-adrenergic receptors (Figure 6). Interestingly, NE application reduced the frequency of sIPSCs recorded in rat LA neurons [45]. This opposing finding to our result in the murine LA may be related to the different species studied. Alternatively, it is feasible that α1-adrenoceptors are particularly involved in the facilitation of inhibitory synaptic transmission in the LA. In line with this notion, α1-adrenergic receptor activity was found to enhance feed-forward inhibition and constrain plasticity related to fear conditioning in the rat LA [46].

Similar to our results reported here, NE was shown previously to facilitate inhibitory synaptic transmission in rat and mouse projection neurons in the BA [35,36,37,38]. Interestingly, Kaneko and coworkers identified a specific subpopulation of GABAergic neurons in the BA of mice which were selectively excited by NE. This effect was mediated via α1-adrenoreceptors and caused increased spontaneous IPSCs in projection neurons [36]. In line with a similar mechanism of NE action in the LA, application of NE did not change sIPSCs in the presence of a α1-adrenoreceptors antagonist (Figure 7). In addition, NE effects were absent after addition of TTX. We conclude that similar to the BA, excitation of LA interneurons by NE leads to increased spiking, thereby causing enhanced frequencies and amplitudes of spontaneous IPSCs in postsynaptic projection neurons.

Importantly, modulation of sIPSCs by NE was strongly reduced in BDNF^+/−^ mice compared to WT littermates. While frequency of sIPSCs was augmented by NE application, amplitudes were enhanced in WT mice only. This variation between genotypes caused a substantial reduction in charge transfer upon NE addition in BDNF^+/−^ mice. Most probably, a specific NE-responsive interneuron subpopulation in the LA is altered in BDNF^+/−^ mice with respect to excitability and/or quantal size or content. In addition, a decrease in the expression of α1-adrenergic receptors [47] could account for our observations.

Indeed, BDNF deficiency seems to especially affect parvalbumin and somatostatin/NPY-expressing interneurons in the cortex [40,48,49,50,51], and somatostatin/NPY-positive interneurons in the amygdala of female mice [52]. Interestingly, different subtypes of LA interneurons were reported to impose unitary IPSCs with unique features onto projection neurons, with stutter-firing interneurons showing the largest amplitudes of unitary IPSCs [53]. In mice, LA interneurons with stutter-firing properties were described to express somatostatin [54] and may represent a GABA interneuron subtype vulnerable to low BDNF function [52]. A detailed characterization of the NE-sensitive interneuron subtype of the LA and its regulation by BDNF remains an important topic of future studies.

### 3.4. Functional Implications

The amygdala receives dense noradrenergic afferents, primarily originating from the locus coeruleus (LC, [55]), which mostly form non-junctional appositions in the LA [56] that typically give rise to volume transmission. During aversive stimuli such as foot shocks, NE release in the amygdala is strongly enhanced [57]. Indeed, release of NE in the amygdala seems to be essential for encoding and retention of memories for emotionally significant events (for recent review, see [58]). The diminished facilitation of sIPSCs by NE in BDNF^+/−^ mice may result in enhanced activation of projection neurons in the amygdala during states of high arousal, and may thereby impair amygdala function [24]. Interestingly, noradrenergic facilitation of GABAergic inhibition was disrupted by chronic stress [35]. Diminished GABAergic tone upon NE action in the amygdala of BDNF^+/−^ mice might therefore enhance stress susceptibility. Importantly, major depressive disorder (MDD), as well as diverse neurodegenerative diseases, are associated with reduced levels of BDNF paralleled by diminished GABAergic neurotransmission [7,59]. The interaction of BDNF and GABA neurotransmission in the amygdala may preferentially involve control of distinct interneuron subtypes by modulatory neurotransmitters, while GABAergic synapses in the LA are not directly modified by chronic BDNF depletion.

## 4. Materials and Methods

### 4.1. Animals

In the present study, male C57BL/6J mice (Charles River, Sulzfeld, Germany) were analyzed. The animals were kept in groups of three to four animals per cage, had free access to food and water, and were maintained at a 12–12 h light-dark-cycle (lights on at 7:00 a.m.). All experiments were carried out in accordance with the European Committees Council Directive (2010/63/EU).

### 4.2. Slice Preparation

Coronal slices were prepared from 4- to 5-week-old male BDNF^+/−^ mice bred on a C57Bl/6J genetic background [60] or their wild-type (WT) littermates, respectively. Mice were decapitated after deep anesthesia with forene (isofluran, 1-Chloro-2,2,2-trifluoroethyl-difluoromethylether) at 4% [61]. A block of tissue containing the amygdala was rapidly removed and transferred into chilled oxygenated saline of the following composition (in mM): NaHCO_3_, 24; KCl, 2.4; MgSO_4_, 10; CaCl_2_, 0.5; NaH_2_PO_4_, 1.25; glucose, 10; sucrose, 195, (pH 7.35). Coronal slices (350 µm thick) were sectioned by a vibratome (Model 1000, The Vibratome Company, St. Louis, MO, USA), and were incubated in standard artificial cerebrospinal fluid (ACFS) containing (in mM): NaCl, 120; KCl, 2.5; NaH_2_PO_4_, 1.25; NaHCO_3_, 24; MgSO_4_, 2; CaCl_2_, 2; glucose, 15; bubbled with 95% O_2_/5% CO_2_ to a final pH of 7.3. Slices were kept at 34 °C for 20 min and for up to 8 h at room temperature. A single slice was then transferred to the recording chamber and submerged in ACSF at a perfusion rate of approximately 2 mL/min at 30 ± 1 °C.

### 4.3. Recording Techniques

Patch clamp recordings were performed in the whole-cell mode on lateral amygdala projection neurons identified by pyramidal-like morphology (EPC-9, Heka, Lamprecht, Germany). Patch pipettes were pulled from borosilicate glass (GC150T-10, Clark Electromedical Instruments, Pangbourne, UK) to resistances of around 3 MΩ when filled with (in mM): Csgluconate, 107; CsCl, 13; MgCl_2_, 1; CaCl_2_, 0.07; EGTA, 11; HEPES, 10; MgATP, 3, Na_3_GTP, 0.5 (pH 7.2 with KOH). A liquid junction potential of 10 mV of the pipette solution was corrected for. After the whole-cell configuration was obtained, neurons were held at 0 mV unless indicated otherwise. Experiments were conducted in the presence of 10 µM 6,7-Dinitroquinoxaline-2,3-dione (DNQX) in combination with 50 µM DL-2-Amino-5-phosphono-pentanoic-acid (AP5) to block glutamatergic synaptic transmission unless indicated otherwise. Norepinephrine (NE) was applied in combination with L-ascorbic acid (40 µM) to reduce degradation.

Inhibitory postsynaptic currents (IPSCs) were evoked by stimuli of 100 µs duration delivered by a stimulus isolator (Isoflex, AMPI, Jerusalem, Israel) at 0.067 Hz. A concentric bipolar electrode (FHC Inc, Bowdoin, ME, USA) was placed on the surface of the slice above the lateral amygdala. Stimulus intensity was adjusted to produce synaptic responses with amplitudes of 400–500 pA to control for similar induction conditions in both genotypes [62]. GABA_A_ receptor mediated miniature IPSCs (mIPSCs) were isolated by addition of 1 µM tetrodotoxin (TTX) to the ACSF. Paired-pulse ratios were derived from two consecutive stimuli separated by interstimulus intervals of 50, 80, and 200 ms in the presence of (2S)-3-[[(1S)-1-(3,4-Dichlorophenyl)ethyl] amino-2-hydroxypropyl] (phenylmethyl) phosphinic acid hydrochloride (CGP-55845, 10 µM). Synaptic fatigue was tested by repetitive stimulation at 40 Hz for 1 s. An average of 2–8 traces was used for analysis.

### 4.4. LTP Recordings

For LTP recordings, ACSF was composed of (in mM): NaCl, 119; KCl, 2.5; NaH_2_PO_4_, 1.25; NaHCO_3_, 26; MgSO_4_, 1; CaCl_2_, 2; glucose, 20; bubbled with 95% O_2_/5% CO_2_. Pipette solution contained (in mM): K-gluconate, 135; KCl, 5; HEPES, 10; MgCl_2_, 2; EGTA, 0.2; MgATP, 4; Na_3_GTP, 0.4; K_3_-phosphocreatine, 10; pH 7.2 with KOH. Neurons were held at −50 mV. The stimulation electrode was placed on the surface of the slice above the LA. LTP was induced within 15 min after whole cell access by pairing focal stimulation (100 Hz, 1 s) with postsynaptic depolarization to −10 mV, two times separated by 20 s. LTP was quantified by normalizing and averaging peak IPSC amplitudes during the last 5 min of experiments (i.e., 30 min after LTP induction) relative to 5 min baseline. Recordings with changes in series resistance above 20% were discarded.

### 4.5. Drugs

Drugs were added to the external ACSF. NE was bath-applied for 5 min and reached its maximal effect around 3 min after application. Slices were pre-incubated with prazosin for 15 to 20 min. All substances were obtained from Sigma-Aldrich (Diesenhofen, Germany), except for DNQX, AP5 and CGP55845 hydrochloride (Tocris, Bristol, UK).

### 4.6. Data Analysis

Data were analyzed with Origin 8.0 (OriginLab Corporation, Northampton, MA, USA). Miniature postsynaptic currents were detected using the program Mini-Analysis (Jaejin software, Leonia, NJ, USA). The rise time of IPSCs was calculated between 10 and 90% of the peak amplitude onset, and the time course of decay was fitted to a mono-exponential function. Cumulative histograms without bins were calculated within time periods of 3 min duration containing exactly 300 events. Release probability (Pr) and synaptic release sites activated by the stimulus (Nsyn) were calculated as described in detail by [18]. In short, the readily releasable pool (RRP) was quantified by back-extrapolating the linear phase of the cumulative amplitude plot of eIPSCs to the y-axis. Pr was calculated as first eIPSCs amplitude divided by the RRP, and Nsyn was given as RRP divided by quantal size. The latter was estimated as the median amplitude of spontaneous IPSCs immediately following 40 Hz stimulation [63].

Statistical analysis was performed using Kolmogorov-Smirnoff (Mini-Analysis) and nonparametric tests by Graph Pad Prism software (San Diego, CA, USA; Wilcoxon signed-rank test for paired observations, Mann–Whitney test for non-paired observations) or by JMP (SAS Institute Inc., Cary, NC, USA, Version 8; analysis of variance (ANOVA) tests, followed by post-hoc Tukey comparisons).

## 5. Conclusions

GABAergic synapses in the LA are neither impaired in pre- nor postsynaptic properties, nor in synaptic plasticity in 4- to 5-week-old BDNF^+/−^ mice, which show around 50% reduced BDNF protein levels in the amygdala. However, facilitation of GABAergic synaptic transmission by NE was significantly decreased in BDNF^+/−^ mice. These findings suggest that BDNF regulates neuromodulation of inhibitory synaptic circuits in the LA, which may become evident during states of high arousal. Chronic BDNF depletion might therefore lead to amygdala dysfunction due to diminished GABAergic tone during emotionally significant events.

## Abbreviations

BA	Basal amygdala
BDNF	Brain-derived neurotrophic factor
BDNF^+/−^	BDNF heterozygous knockout mice
LA	Lateral amygdala
iLTP	Inhibitory long-term potentiation
LTP	Long-term potentiation
NE	Norepinephrine
TrkB	Tropomyosin-related kinase B
